# Social protection spending and inequalities in depressive symptoms across Europe

**DOI:** 10.1007/s00127-016-1223-6

**Published:** 2016-04-30

**Authors:** Claire L. Niedzwiedz, Richard J. Mitchell, Niamh K. Shortt, Jamie R. Pearce

**Affiliations:** Centre for Research on Environment, Society and Health, University of Edinburgh, Drummond Street, Edinburgh, EH8 9XP Scotland, UK; Centre for Research on Environment, Society and Health, University of Glasgow, 1 Lilybank Gardens, Glasgow, G12 8RZ Scotland, UK

**Keywords:** Inequality, Depression, Socioeconomic factors, Europe, Employment

## Abstract

**Purpose:**

Common mental disorders are an increasing global public health concern. The least advantaged in society experience a greater burden of mental illness, but inequalities in mental health vary by social, political, and economic contexts. This study investigates whether spending on different types of social protection alters the extent of social inequality in depressive symptoms.

**Methods:**

Data were obtained from the 2006 and 2012 cross-sectional waves of the European Social Survey, which included 48,397 individuals from 18 European countries. Depressive symptoms were measured using the Centre for Epidemiologic Studies-Depression Scale (CES-D 8). Statistical interactions between country-level social protection spending and individuals’ education level, employment and family status were explored using multilevel regression models.

**Results:**

Higher spending on active labour market programmes was related to narrower inequality in depressive symptoms by education level. Compared to men with high education, the marginal effect of having low education was 1.67 (95 % CI, 1.46–1.87) among men in countries with lower spending and 0.85 (95 % CI, 0.66–1.03) in higher spending countries. Single parents exhibited fewer depressive symptoms, as spending on family policies increased. Little evidence was found for an overall association between spending on unemployment benefits and employment-related inequalities in depressive symptoms, but in 2012, unemployment spending appeared beneficial to mental health among the unemployed.

**Conclusions:**

Greater investment in social protection may act to reduce inequalities in depressive symptoms. Reductions in spending levels or increased conditionality may adversely affect the mental health of disadvantaged social groups.

**Electronic supplementary material:**

The online version of this article (doi:10.1007/s00127-016-1223-6) contains supplementary material, which is available to authorized users.

## Introduction

Common mental disorders, such as depression, are sensitive to the social, political, and economic environments in which people live. The recent global financial crisis, for example, has demonstrated that changes to the unemployment rate and welfare system can have a significant impact on population mental health, as demonstrated by increased depression [1, 2] and suicide rates [3–5] across several countries. Like many health conditions, depression is socially patterned; the least advantaged in society experience poorer mental health [6–8]. Gender also contributes with women reporting poorer mental health compared to men across the socioeconomic gradient [6], which may be due to the unequal distribution of power between men and women, as well as between the least and most educated individuals. Feeling a lack of control over one’s life is an important social determinant of health [9] and risk factor for depression [10], and powerlessness related to the traditional gender roles of employment, care-giving, and housekeeping may exacerbate psychological distress [11]. Furthermore, the impact of unemployment on mental health is stronger for men compared to women, but likely influenced by family responsibilities [12]. The larger association between unemployment and poor mental health among men is thought to be related to the greater financial strain, sense of social status, social support, and self-esteem; they obtain from paid work, compared to women [12, 13]. Research has also demonstrated that the relationship between unemployment and psychological distress differs depending on family status; having children appears to be protective for unemployed women, but may exacerbate poor mental health among unemployed men, with little differences found in terms of the duration of unemployment [12]. However, the extent to which social and economic factors are associated with mental health and wellbeing is not consistent across different societies [14]. This suggests that the features of the political and economic systems may moderate the influence of individual-level factors on mental health. These concerns are particularly pertinent during a period of rapid change in welfare policy across several countries that have followed the global financial crisis.

A body of the literature suggests that the social inequalities in mental health and wellbeing vary according to the type of welfare state, or ‘welfare regime’ under consideration [15–19]. The welfare regime approach to study the effects of welfare policy on inequalities in health and wellbeing is based on the assumption that welfare states cluster into distinct regimes according to their similar social policies, political traditions, and ideologies, which tend to remain stable over time [19, 20]. For example, the Scandinavian or Nordic welfare regime has traditionally been defined by more generous and universal welfare benefits, as well as other characteristics, such as full employment [21]. Several studies have demonstrated that the social inequalities in mental health and wellbeing are smaller in welfare regimes considered more egalitarian, such as the Nordic countries, but others have demonstrated inconsistent results [15, 20, 22]. Key criticisms of the approach taken in this research are the inability to uncover specific policies that may help to reduce the social inequalities in mental health and wellbeing, and the categorisation of countries into the same regime that sometimes have quite distinct policies [23]. Studies have, therefore, sought to improve on this approach by examining how health, wellbeing, and inequalities vary depending on the level of investment in social protection policies [23–25].

Government investment in social protection may act to reduce financial strain and psychosocial stress among the most disadvantaged in society [26], and could, therefore, be important in helping to reduce the inequalities in mental health. Investment in social protection aims to guard against various social risks, such as those related to unemployment, single parenthood, or disability. Protection is provided in the form of cash benefits or in-kind resources, the latter, including goods and services, such as training opportunities provided through active labour market programmes (ALMPs), or early childhood education provided by family-based policies. Relatively few studies have examined the relationship between social protection and social inequalities in mental health. It could be hypothesised that the most disadvantaged in society, for example, those who are unemployed or have few educational qualifications, benefit more from more generous levels of social protection, as financial and psychosocial stress may be reduced.

To identify potential policy opportunities for reducing inequalities in mental health, this paper examines the role of different levels of spending on various types of social protection in moderating the extent of social inequality in depressive symptoms across 18 European countries. We also examine whether the potential moderating effects have changed between 2006 and 2012. The response to the recent global financial crisis has seen the implementation of austerity measures across much of Europe that have reduced investment in social protection programmes [27]. It could, therefore, be hypothesised that spending on social protection policies, such as unemployment benefits and work activation programmes, may have become more important for the mental health of disadvantaged groups, as unemployment has increased and disposable income decreased [28].

## Methods

### Study sample

Individual-level data were taken from the third 2006/07 (edition 3.5) [29] and sixth 2012/13 (edition 2.0) [30] waves of the European Social Survey (ESS). These rounds were selected, as depressive symptoms were only measured at the two time points. The ESS is a cross-sectional survey conducted every 2 years and is the representative of individuals aged 15 years and over resident in private households in each country, regardless of nationality, citizenship, or language. Individuals were selected by strict random probability methods at every stage [31]. Response rates varied from 46.0 % in France to 73.2 % in Slovakia in the 2006/07 round and from 33.8 % in Germany to 77.1 % in Portugal in the 2012/13 round [32]. We included individuals aged 20–64 years to represent the working-age population [33]. Country-level data were taken from the Organisation for Economic Co-operation and Development (OECD) and Eurostat. All country-level data correspond to the year before ESS data collection (either 2005 or 2011); this was mainly due to the absence of 2012 social protection data. We included data from 18 countries (Belgium, Switzerland, Germany, Denmark, Estonia, Spain, Finland, France, United Kingdom, Hungary, Ireland, Netherlands, Norway, Poland, Portugal, Sweden, Slovenia, and Slovakia) for which data were available for both the waves of the ESS and were included in the OECD Social Expenditure (SOCX) database [34].

### Measures

Depressive symptoms were measured using the Center for Epidemiologic Studies-Depression Scale (CES-D 8); a shortened version of the 20-item CES-D that is used to assess the symptoms of depression in the general population [35]. CES-D 8 is a validated self-report questionnaire which asks participants how much of the time during the past week: (1) felt depressed, (2) felt everything was an effort, (3) had restless sleep, (4) were happy, (5) felt lonely, (6) enjoyed life, (7) felt sad, (8) felt unable to get going [36]. The response categories were none or almost none of the time, some of the time, most of the time, or all or almost all of the time. The scale ranges from 0 to 24; higher scores indicate higher depressive symptoms and heightened risk of clinical depression [37]. The outcome was treated as continuous, because no clear cut-off has been described for identifying potential depression ‘cases’, and previous studies have analysed the scale using linear models [14].

Social inequality in depressive symptoms was considered according to three socio-demographic variables: education level, employment status, and family status. Participants’ highest education level was recorded using the International Standard Classification of Education (ISCED) [38] and divided into low (less than lower secondary education, or lower secondary education completed), medium (upper secondary education or post-secondary non-tertiary education completed), and high (tertiary education completed). Employment status was assessed by asking respondents about their activity in the past 7 days and categorised into employed, unemployed, permanently sick or disabled, or other (including those who were looking after the home or family, undertaking community or military service, and retired or in education). Family status was derived from respondents’ marital, cohabitation, and parental status and divided into those who were married/cohabiting with children, married/cohabiting without children, single (never married, widowed, divorced, and living alone) with children, or single without children. Age and immigrant status (categorised as those who were born in their country of residence or not) were considered as potential confounding variables. An age-squared term was also included as a non-linear association between age and CES-D 8 scores was apparent.

Disaggregated country-level public expenditure on social protection was extracted from the OECD SOCX database. We included three types of social expenditure that we hypothesised which were likely to moderate specific inequalities in depressive symptoms: unemployment, active labour market programmes (ALMPs), and family (see Online Resource Table S1 for further detail). Spending on family policies was measured in US dollars per head at 2005 constant prices and purchasing Power Parity (PPP). We hypothesised that greater spending on family policies (such as investment in early childhood education and care) may help to reduce the symptoms of depression particularly among single parents perhaps via reducing financial and psychosocial strain, but also recognise that the investment in these policies may also benefit coupled families with children. For spending on unemployment and ALMPs, we calculated the total spent per person unemployed by multiplying spending per head of population by the total working-age population and dividing by the number of people who were unemployed during the respective years. Spending on unemployment was hypothesised to reduce the mental health burden on those who were unemployed, perhaps by reducing the financial strain associated with being out of work. Investment in ALMPs was considered to help reduce the inequalities in mental health by education level, and it was hypothesised that the least educated groups would benefit more from higher spending. This might be plausible, because the least educated groups, at higher risk of unemployment, may profit more from programmes and training that help to build their skills and confidence, and, therefore, increase their chances of finding, and staying in, work. We might also expect those with fewer educational qualifications to benefit more from investment in ALMPs, regardless of employment status, as they may feel less concerned knowing that there is support available to assist in re-employment if needed. The social protection spending variables were converted to z-scores to allow direct comparisons to be made. GDP per head in US dollars at constant PPPs and prices was also included as a potential country-level confounding variable.

### Statistical analysis

Descriptive statistics for each individual- and country-level variable were first examined, followed by mean depressive symptoms according to the three socio-demographic variables of interest. To first examine whether the association between employment status, education level, and family status, and depressive symptoms varied by country, we calculated single-level linear regression models with interactions between the country dummy variables and the socio-demographic variables (controlling for age, age-squared, and immigrant status). Evidence for statistical interactions was investigated using Wald tests. Random-intercept multilevel linear regression models were then calculated, which included individuals nested within countries. To examine the potential moderating effects of social protection spending on inequalities in depressive symptoms, a series of models were calculated. For each socio-demographic variable examined, first, the individual-level variables (age, age-squared, immigrant status, and the socio-demographic variable of interest) and the survey year were entered into the models, followed by the country-level variables (social protection spending, GDP per capita). Then, interactions between the socio-demographic and social protection spending variables were tested. We additionally tested the interaction between the survey year, spending on social protection, and the socio-demographic variables to examine whether associations differed between years. Average marginal effects on CES-D 8 scores for a one standard deviation (SD) increase and decrease in social protection spending across the different social groups were calculated, and marginal mean predicted depressive symptoms were plotted by social group according to different levels of social protection spending to aid the interpretation of interactions.

All analyses were stratified by gender, as we hypothesised that relationships may differ by sex and interactions were statistically significant. All models examining inequalities in depressive symptoms by employment status controlled for education level and family status. Models investigating family status controlled for education level. In addition, we performed a sensitivity analysis including employment status in the models, examining the interaction between education level and spending on ALMPs. The sample included 50,003 individuals; those with missing exposure or outcome data were excluded (Online Resource Table S2). Analyses were performed using Stata/MP 12.1.

## Results

### Description of the sample

48,397 individuals (52.49 % female, mean age = 42.88, and SD = 12.60) from 18 countries were included in the analyses (Table 1), after excluding those with missing data (*N* = 1606, 3.21 %). Individuals with missing outcome data were more likely to be older, less educated, out of work, single with no children, and born outside their current country of residence. The percentage of the variance in depressive symptoms explained by the country level was 5.88 % (95 % CI, 3.12–10.81) among men and 5.17 % (95 % CI, 2.74–9.57) for women.

**Table 1:** Mean CES-D 8 scores by gender and country

	Men			Women		
	Mean	SD	*N*	Mean	SD	*N*
Belgium	4.65	3.63	1262	5.69	4.17	1347
Switzerland	4.27	3.28	1132	5.04	3.52	1233
Germany	5.45	3.40	2021	6.04	3.90	2005
Denmark	4.44	2.99	1089	4.85	3.49	1079
Estonia	6.06	3.86	1125	6.22	3.87	1395
Spain	5.11	3.91	1329	6.14	4.39	1350
Finland	4.55	3.02	1467	4.63	3.35	1383
France	4.90	3.80	1317	6.07	4.54	1524
UK	5.36	4.04	1371	6.04	4.35	1757
Hungary	7.64	4.66	1099	8.01	4.68	1341
Ireland	4.82	4.00	1398	4.71	3.88	1653
Netherlands	4.66	3.53	1266	5.41	3.89	1444
Norway	3.87	2.99	1353	4.27	3.17	1188
Poland	5.20	4.14	1248	6.34	4.95	1291
Portugal	5.99	3.92	1148	7.20	4.53	1728
Sweden	4.33	3.40	1362	5.17	4.00	1314
Slovenia	4.60	3.25	859	5.05	3.78	997
Slovakia	6.70	3.61	1145	6.96	3.87	1377
Total	5.12	3.75	22,991	5.82	4.17	25,406

The overall mean spending per head across countries and years on family-related policies was $764.76 (SD = 381.15), and the mean spent per person unemployed was $8937.33 (SD = 7269.67) for unemployment benefits and $5852.31 (SD = 4658.79) for ALMPs (see Online Resource Table S3 for a breakdown by country). The key results for the statistical models are reported below, with full results available in the Online Resources.

### Overall associations

Compared to employed individuals, all other employment status groups had a higher level of depressive symptoms (Online Resource Table S4 Models 1). Those who were permanently sick or disabled had the greatest level of symptoms, followed by the unemployed, and the ‘other’ categories. The association between unemployment and depressive symptoms was also stronger for men (*b* = 1.72, 95 % CI, 1.55–1.89) compared to women (1.34, 95 % CI, 1.15–1.54). There was an educational gradient in depressive symptoms; the least educated experienced higher depressive symptoms compared to the most educated, and the extent of inequality in depressive symptoms was larger among women compared to men (Online Resource Table S5 Models 1). Differences in the level of depressive symptoms were also apparent by family status and varied by gender. Compared to men who were married or cohabiting with children, men who were married/cohabiting but did not have children had slightly higher depressive symptoms, but no difference was found among women (Online Resource Table S6 Models 1). However, the highest level of depressive symptoms was found among men and women who were single parents. Those who were single and had no children also experienced higher levels of depressive symptoms compared to parents who were married or cohabiting. There was evidence to suggest that the association between the three socio-demographic variables and depressive symptoms varied by country, as all Wald tests were statistically significant (*p* < 0.001, results available on request).

### Spending on unemployment

**Fig. 1:**
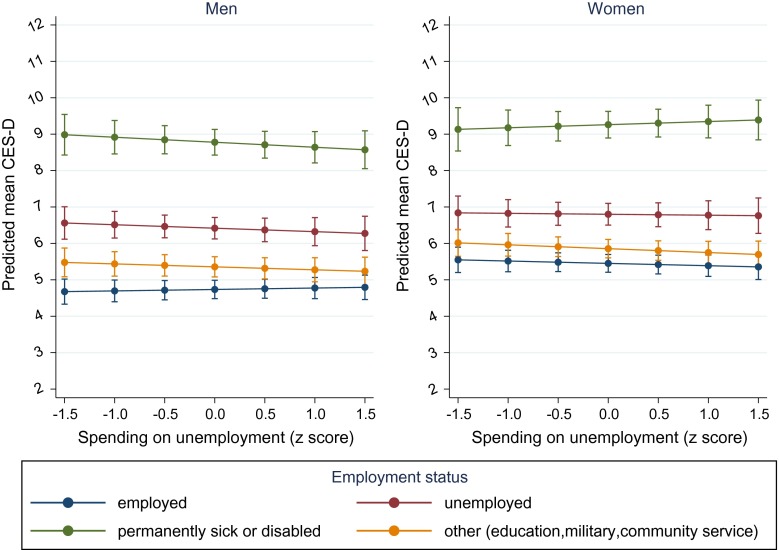
Marginal predicted mean depressive symptoms (CES-D 8) by employment status and spending on unemployment, with 95 % confidence intervals

Among men, as spending on unemployment policies increased, depressive symptoms tended to decrease among all employment status groups except the employed (Online Resource Table S4), but the results were not statistically significant (Fig. 1). However, in 2012, there was evidence to suggest that increased spending on unemployment benefits was related to fewer depressive symptoms among the unemployed. Spending on unemployment benefits did not appear to moderate the influence of employment status on depressive symptoms among women.

### Spending on active labour market programmes (ALMPs)

**Fig. 2:**
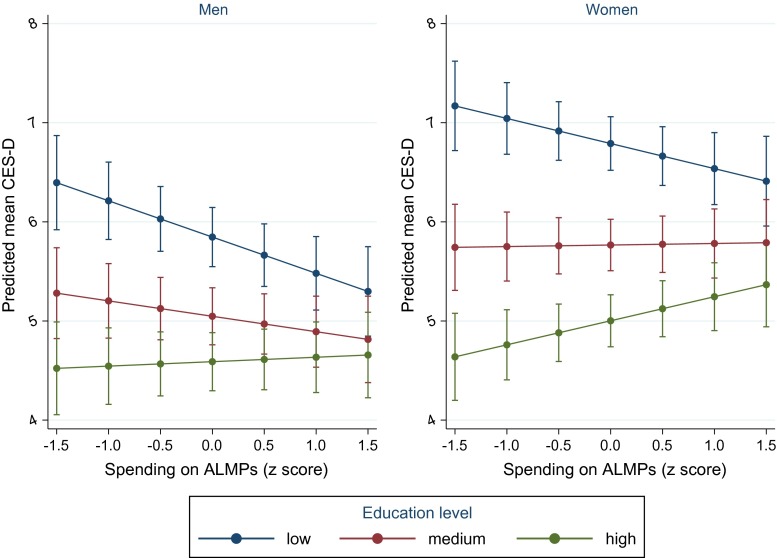
Marginal predicted mean depressive symptoms (CES-D 8) by educational level and spending on ALMPs with 95 % confidence intervals

Increased spending on ALMPs was related to a narrowing of educational inequality in depressive symptoms. For men, this appeared to be driven by decreased symptoms among the least educated groups (Fig. 2). The marginal effect of low education, compared to high education, on depressive symptoms was 1.67 (95 % CI, 1.46–1.87) among men in countries with lower ALMP spending (one SD below the mean) and 0.85 (95 % CI, 0.66–1.03) in those with higher spending (one SD above the mean) (Table 2). Among women, the equivalent results were 2.28 (95 % CI, 2.08–2.48) in lower spending countries and 1.29 (95 % CI, 1.09–1.49) in higher spending countries. Results were consistent across years, although there was a suggestion that in 2012, the association between ALMP spending and depressive symptoms among the least educated women was weaker than in 2006 (Online Resource Table S5). Including employment status in the models made little difference to the results (Online Resource Table S6).

**Table 2:** Average marginal effects on depressive symptoms at different levels of social protection spending

	Spending level	Unemployment spending: comparing unemployed with employed		ALMP spending: comparing low education with high education		Family spending: comparing those single with children to married/cohabiting with children	
		Men	Women	Men	Women	Men	Women
		Marginal effect [95 % CI]	Marginal effect [95 % CI]	Marginal effect [95 % CI]	Marginal effect [95 % CI]	Marginal effect [95 % CI]	Marginal effect [95 % CI]
Average marginal effect on depressive symptoms	1 SD below mean	1.82 [1.58, 2.05]	1.31[1.05, 1.57]	1.67 1[1.46, 1.87]	2.28 [2.08, 2.48]	2.51 [2.00, 3.02]	1.86 [1.62, 2.09]
	1 SD above mean	1.55 [1.28, 1.82]	1.39 [1.10, 1.68]	0.85 [0.66, 1.03]	1.29 [1.09, 1.49]	1.54 [1.13, 1.95]	1.31 [1.07, 1.54]

*ALMP* active labour market programmes, *CI* confidence interval, *SD* standard deviation

### Spending on family

**Fig. 3:**
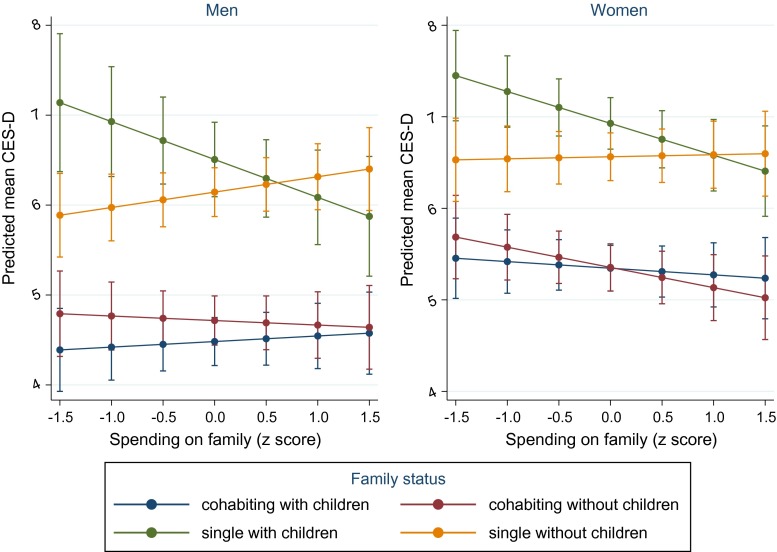
Marginal predicted mean depressive symptoms (CES-D 8) by family status and spending on family policies with 95 % confidence intervals

Higher spending on family-related policies was related to fewer depressive symptoms among single parents (Fig. 3). Compared to those who were married/cohabiting and had children, the marginal effect of being single with children was 2.51 (95 % CI, 2.00–3.02) among men and 1.86 (95 % CI, 1.62–2.09) among women in countries with lower spending (one SD below the mean) and 1.54 (95 % CI, 1.13–1.95) and 1.31 (95 % CI, 1.07–1.54) among men and women in higher spending countries (one SD above the mean), respectively (Table 2). Increased spending also appeared to amplify depressive symptoms among men who were single and did not have children and to a lesser extent among women, but the associated slopes were not significantly different to those for people who were married/cohabiting and had children (Online Resource Table S7). The relationships were reasonably consistent across both years.

## Discussion

Our results demonstrate that different forms of social protection spending may have a role in moderating the extent of social inequality in depressive symptoms across Europe. We found greater spending in specific areas of welfare policy was associated with fewer depressive symptoms among disadvantaged social groups, especially those with the least education and single parents. There was little evidence that social protection spending had a substantially greater moderating role following the recent economic recession in Europe, with results reasonably consistent across years. This could be because the full extent of cuts to social protection spending had not been fully realised in 2012. There is also evidence to suggest that, at least in some countries, such as the United Kingdom, the nature of social protection support is changing, with increasing ‘conditionality’, including more stringent eligibility criteria and sanctions for non-compliance, which may be damaging to mental health [39].

Greater spending on ALMPs was associated with a narrower education gradient in depressive symptoms among both genders. ALMPs may help to reduce depressive symptoms among the least educated groups, who are more likely to become unemployed, by increasing skill acquisition and restoring a sense of purpose, which could aid return-to-work and prevent future unemployment. The decreased education-related inequality in depressive symptoms among men appeared to be driven via reduced depressive symptoms among the least educated group. However, among women and to a lesser extent among men, there was a suggestion that increased spending on ALMPs may be related to increased symptoms among the highest educated, perhaps because this group knows that they are likely to experience little benefit from such programmes and in countries investing more, those with the highest education may feel that they are losing out whilst those with the least education receive more investment. Higher spending on family policies was related to fewer depressive symptoms among both single men and women with children living in their household. This suggests that policies, such as parental leave, child allowances, and early childhood education, could help to relieve the strain of competing demands relating to work and family, as well as the financial burden, which may reduce depressive symptoms, such as restless sleep and feeling like everything, is an effort. However, it should be stressed that these are only hypotheses and further research is recommended to uncover the potential pathways underlying the results. Additional research is needed to confirm the generalisability of our results to other countries, such as the US, where advantage can be taken of the varying generosity of welfare programmes between states [4].

Previous research has demonstrated the importance of ALMPs in moderating the relationship between unemployment and male suicide [3], which is consistent with our results demonstrating ALMPs may reduce educational inequality in depressive symptoms. The same study also found no relationship between spending on the unemployment benefits and the unemployment–suicide relationship. More generous unemployment benefits have been related to higher subjective wellbeing among both employed and unemployed individuals [40], which is in contrast to our finding that the generosity of benefits is not consistently related to the mental health of the unemployed. However, it is possible that the social factors which influence positive mental health are different to those for depressive symptoms [41]. Greater spending on unemployment benefits across the US was also found to reduce the negative impact of higher unemployment on suicide rates [4], and more generous unemployment benefits have been related to decreased psychological distress among the unemployed [42]. However, a key weakness of the latter study was the lack of comparable cross-national data and the different operationalisation of social protection variables make the results difficult to compare. In general terms, our results concur with those studies finding that the more egalitarian welfare regimes, which include countries, such as Denmark and the Netherlands, who tend to spend more on policies, such as ALMPs, have narrower inequalities in mental health [16, 18, 43].

Our paper has a number of strengths, including the use of cross-nationally comparable data and a validated measure of depressive symptoms. The examination of inequalities in depressive symptoms by several different socio-demographic variables and the moderating influence of disaggregated social protection expenditure is also an improvement on previous research. However, the limitations of our paper should be acknowledged. The restricted number of countries included may have affected the statistical power of the models. We were also limited by the lack of available data on depressive symptoms during the peak recession period and the cross-sectional design of the survey also restricts our ability to infer causality. Thus, longitudinal studies which examine changing levels of social protection and individual changes in depressive symptoms and other common mental health outcomes are needed. We also cannot rule out the possibility of residual confounding, particularly with regard to whether other types of social protection we did not investigate may be confounding the relationships. Countries with higher spending on social protection policies potentially also share other characteristics (such as higher social capital) that help to relieve depressive symptoms among more disadvantaged groups. Our measure of unemployment is also limited to activities in the past 7 days and, therefore, does not account for the duration of unemployment.

## Conclusions

Our findings suggest that decisions relating to the levels of investment in social protection could have important implications for the mental health of different social groups, particularly those considered socially disadvantaged. However, associations may vary depending on the type of social protection. Countries that invest more in specific types of social protection, such as ALMPs, could be considered to be equigenic [44], reducing the extent of socioeconomic inequality in mental health. Therefore, our results have potentially important implications for policy and practise, but recognise that additional research is required to further investigate whether causal effects are likely. It has been argued that depressive symptoms are related to psychosocial and functional impairment, even when below the threshold for a clinical diagnosis of depression [45]. Therefore, our findings are of potential clinical significance and raise the possibility that social protection spending has unintended effects on inequalities in mental health. Reductions to social protection brought about by regressive austerity measures may have potentially damaging effects on the mental health of particular groups, such as those with lower education and single parents. This could not only have deleterious impact on mental health, which may increase the burden on health services, but it could also impact on societal outcomes over the longer term. Employment rates and economic growth may be affected, as those with poorer mental health may be less likely to be employed [46]. Therefore, it is imperative that further research explores changes to the levels of social protection spending and changes in population mental health and inequalities, from a casual perspective and that research continues into the optimal level of investment in social protection which benefits public health and health inequalities, especially given the heavy economic and societal costs of health inequalities. Policy-makers should also fully consider the mental health impact and associated costs to society that any change to social protection spending might produce.

## Electronic supplementary material

Below is the link to the electronic supplementary material.

Table S1: Types of social protection spending and related hypotheses. Table S2: Frequencies of key variables and extent of missing data. Table S3: Summary of country-level variables. Table S4: Linear multilevel models predicting depressive symptoms (CES-D 8) according to employment status and unemployment spending among men and women from 18 European countries. Table S5: Linear multilevel models predicting depressive symptoms (CES-D 8) according to education level and ALMP spending among men and women from 18 European countries. Table S6: Linear multilevel models predicting depressive symptoms (CES-D 8) according to education level and ALMP spending among men and women from 18 European countries, controlling for employment status. Table S7: Linear multilevel models predicting depressive symptoms (CES-D 8) according to family status and family spending among men and women from 18 European countries
