# Supplementary material for: Social protection spending and inequalities in depressive symptoms across Europe
Source: Soc Psychiatry Psychiatr Epidemiol. 2016 Apr 30;51:1005–14. doi: 10.1007/s00127-016-1223-6 (PMC4947487; doi:10.1007/s00127-016-1223-6)
Supplement: Supplementary file 1 — Table S1: Types of social protection spending and related hypotheses. Table S2: Frequencies of key variables and extent of missing data. Table S3: Summary of country-level variables. Table S4: Linear multilevel models predicting depressive symptoms (CES-D 8) according to employment status and unemployment spending among men and women from 18 European countries. Table S5: Linear multilevel models predicting depressive symptoms (CES-D 8) according to education level and ALMP spending among men and women from 18 European countries. Table S6: Linear multilevel models predicting depressive symptoms (CES-D 8) according to education level and ALMP spending among men and women from 18 European countries, controlling for employment status. Table S7: Linear multilevel models predicting depressive symptoms (CES-D 8) according to family status and family spending among men and women from 18 European countries [file 127_2016_1223_MOESM1_ESM.docx]

Electronic supplementary material

**Social protection spending and inequalities in depressive symptoms across Europe**

**Social Psychiatry and Psychiatric Epidemiology**

^1*^ Claire L Niedzwiedz, PhD

^2^ Richard J Mitchell, PhD

^1^ Niamh K Shortt, PhD

^1^ Jamie R Pearce, PhD

^1^ Centre for Research on Environment, Society and Health, University of Edinburgh, Drummond Street, Edinburgh, Scotland, EH8 9XP

^2^ Centre for Research on Environment, Society and Health, University of Glasgow, 1 Lilybank Gardens, Glasgow, Scotland, G12 8RZ

^*^ Corresponding author: Claire L Niedzwiedz (claire.niedzwiedz@gmail.com).

**Contents**

Table S1: Types of social protection spending and related hypotheses

Table S2: Frequencies of key variables and extent of missing data

Table S3: Summary of country-level variables

Table S4: Linear multilevel models predicting depressive symptoms (CES-D 8) according to employment status and unemployment spending among men and women from 18 European countries

Table S5: Linear multilevel models predicting depressive symptoms (CES-D 8) according to education level and ALMP spending among men and women from 18 European countries

Table S6: Linear multilevel models predicting depressive symptoms (CES-D 8) according to education level and ALMP spending among men and women from 18 European countries, controlling for employment status

Table S7: Linear multilevel models predicting depressive symptoms (CES-D 8) according to family status and family spending among men and women from 18 European countries

Table S1: Types of social protection spending and related hypotheses

| **Type of social protection spending** | **Unemployment** | **Active Labour Market Programmes (ALMPs)** | **Family** |
| --- | --- | --- | --- |
| **Components** | - Unemployment compensation /severance pay - Early retirement for labour market reasons | - Public employment service and administration - Training - Job rotation and job sharing - Employment incentives - Supported employment and rehabilitation - Direct job creation - Start-up incentives | - Family allowances - Maternity and parental leave - Other cash benefits - Early childhood education and care - Home help/accommodation - Other benefits in kind |
| **Type of social inequality examined** | Employment status | Education level | Family status |
| **Hypotheses** | Spending on unemployment policies may reduce depressive symptoms among the unemployed | Spending on ALMPs may reduce depressive symptoms among the least educated | Spending on family-related policies may help to reduce depressive symptoms among single parents |

SD=standard deviation

Table S2: Frequencies of key variables and extent of missing data

| **Education Level** | **N** | **%** |
| --- | --- | --- |
| Low | 11,404 | 22.81 |
| Medium | 22,524 | 45.05 |
| High | 15,717 | 31.43 |
| Missing | 358 | 0.72 |
| **Gender** |  |  |
| Male | 23,751 | 47.50 |
| Female | 26,224 | 52.44 |
| Missing | 28 | 0.06 |
| **Employment status** | |  |
| Employed | 33,028 | 66.05 |
| Unemployed | 3,846 | 7.69 |
| permanently sick or disabled | 1,601 | 3.20 |
| other (education, military, community service) | 11,247 | 22.49 |
| Missing | 281 | 0.56 |
| **Immigrant status** | |  |
| born in country | 44,942 | 89.88 |
| born outside country | 5,026 | 10.05 |
| Missing | 35 | 0.07 |
| **Family status** | |  |
| married/cohabiting with children | 20,516 | 41.03 |
| married/cohabiting without children | 12,768 | 25.53 |
| single with children | 3,238 | 6.48 |
| single without children | 13,232 | 26.46 |
| Missing | 249 | 0.50 |
| **CES-D 8** |  |  |
| not missing | 49,160 | 98.31 |
| Missing | 843 | 1.69 |
| Total | 50,003 | 100.00 |

Table S3: Summary of country-level variables

|  | **Unemployment spending**^1^ | **ALMP spending**^1^ | **Family spending**^2^ | **GDP**^3^ |
| --- | --- | --- | --- | --- |
| Belgium | 26331.32 | 5957.91 | 904.41 | 335.76 |
| Switzerland | 9896.86 | 8259.80 | 474.86 | 402.87 |
| Germany | 7959.35 | 4842.70 | 633.47 | 339.21 |
| Denmark | 22793.10 | 16089.12 | 1282.70 | 338.50 |
| Estonia | 620.40 | 434.89 | 409.75 | 174.48 |
| Spain | 8598.43 | 3046.67 | 362.15 | 275.70 |
| Finland | 12157.23 | 6413.32 | 959.95 | 327.50 |
| France | 9224.47 | 5118.77 | 872.65 | 307.67 |
| UK | 3340.62 | 4719.20 | 1168.71 | 344.64 |
| Hungary | 2772.88 | 1443.15 | 665.36 | 174.46 |
| Ireland | 12656.47 | 6262.39 | 1347.36 | 387.17 |
| Netherlands | 18839.64 | 14994.89 | 586.80 | 382.49 |
| Norway | 9753.91 | 13472.00 | 1359.74 | 472.40 |
| Poland | 823.28 | 1099.96 | 204.38 | 160.28 |
| Portugal | 4353.92 | 2116.96 | 258.06 | 221.49 |
| Sweden | 6387.34 | 9334.59 | 1195.59 | 353.29 |
| Slovenia | 4045.65 | 2030.84 | 574.92 | 246.55 |
| Slovakia | 1046.10 | 718.25 | 403.36 | 190.08 |
| **Mean** | 8937.33 | 5852.31 | 764.76 | 305.02 |

^1^ US dollars per person unemployed, at constant prices (2005) and constant PPPs (2005) from OECD ^2^ US dollars per head, at constant prices (2005) and constant PPPs (2005) from OECD; ^3^ Gross domestic product (expenditure approach) US dollars per head, constant prices, constant PPPs, OECD base year (2005)

**Table S4: Linear multilevel models predicting depressive symptoms (CES-D 8) according to employment status and unemployment spending among men and women from 18 European countries**

|  | **Men** | | | | **Women** | | | |
| --- | --- | --- | --- | --- | --- | --- | --- | --- |
|  | **Model 1** | **Model 2** | **Model 3** | **Model 4** | **Model 1** | **Model 2** | **Model 3** | **Model 4** |
|  | **b**  **[95% CI]** | **b**  **[95% CI]** | **b**  **[95% CI]** | **b**  **[95% CI]** | **b**  **[95% CI]** | **b**  **[95% CI]** | **b**  **[95% CI]** | **b**  **[95% CI]** |
| Age (centered) | 0.02^***^ [0.01,0.02] | 0.02^***^ [0.01,0.02] | 0.02^***^ [0.01,0.02] | 0.02^***^ [0.01,0.02] | 0.02^***^ [0.02,0.03] | 0.02^***^ [0.02,0.03] | 0.02^***^ [0.02,0.03] | 0.02^***^ [0.02,0.03] |
| Age squared | -0.22^***^ [-0.25,-0.18] | -0.22^***^ [-0.25,-0.18] | -0.22^***^ [-0.25,-0.18] | -0.22^***^ [-0.25,-0.18] | -0.13^***^ [-0.17,-0.09] | -0.13^***^ [-0.17,-0.09] | -0.13^***^ [-0.17,-0.09] | -0.13^***^ [-0.17,-0.09] |
| Not born in country^a^ | 0.81^***^ [0.66,0.97] | 0.81^***^ [0.66,0.97] | 0.82^***^ [0.67,0.98] | 0.82^***^ [0.67,0.98] | 0.77^***^ [0.61,0.93] | 0.77^***^ [0.61,0.93] | 0.77^***^ [0.60,0.93] | 0.76^***^ [0.60,0.92] |
| Married/cohabiting without children^b^ | 0.16^**^ [0.04,0.28] | 0.16^**^ [0.04,0.28] | 0.16^**^ [0.04,0.28] | 0.16^**^ [0.04,0.28] | -0.05 [-0.18,0.09] | -0.04 [-0.18,0.09] | -0.04 [-0.17,0.09] | -0.04 [-0.17,0.10] |
| Single with children^b^ | 1.73^***^ [1.42,2.05] | 1.73^***^ [1.42,2.05] | 1.73^***^ [1.41,2.04] | 1.72^***^ [1.41,2.04] | 1.49^***^ [1.32,1.66] | 1.49^***^ [1.32,1.66] | 1.49^***^ [1.32,1.66] | 1.49^***^ [1.32,1.65] |
| Single without children^b^ | 1.33^***^ [1.21,1.45] | 1.33^***^ [1.21,1.45] | 1.33^***^ [1.21,1.45] | 1.33^***^ [1.21,1.45] | 1.07^***^ [0.93,1.21] | 1.07^***^ [0.93,1.21] | 1.07^***^ [0.93,1.21] | 1.07^***^ [0.93,1.21] |
| Low education^c^ | 0.72^***^ [0.59,0.86] | 0.72^***^ [0.58,0.85] | 0.72^***^ [0.59,0.85] | 0.72^***^ [0.59,0.86] | 1.43^***^ [1.29,1.57] | 1.43^***^ [1.29,1.57] | 1.43^***^ [1.29,1.57] | 1.43^***^ [1.29,1.57] |
| Medium education^c^ | 0.25^***^ [0.14,0.36] | 0.24^***^ [0.13,0.35] | 0.24^***^ [0.13,0.35] | 0.24^***^ [0.13,0.35] | 0.60^***^ [0.48,0.71] | 0.59^***^ [0.48,0.71] | 0.59^***^ [0.48,0.71] | 0.59^***^ [0.48,0.71] |
| 2012 survey year^d^ | -0.34^***^ [-0.43,-0.25] | -0.24^***^ [-0.34,-0.14] | -0.24^***^ [-0.34,-0.14] | -0.23^***^ [-0.34,-0.11] | -0.37^***^ [-0.47,-0.28] | -0.30^***^ [-0.41,-0.20] | -0.31^***^ [-0.41,-0.20] | -0.29^***^ [-0.42,-0.15] |
| Unemployed^e^ | 1.72^***^ [1.55,1.89] | 1.70^***^ [1.53,1.87] | 1.68^***^ [1.51,1.85] | 1.82^***^ [1.55,2.10] | 1.36^***^ [1.16,1.55] | 1.34^***^ [1.15,1.54] | 1.35^***^ [1.15,1.54] | 1.48^***^ [1.17,1.78] |
| Permanently sick/disabled^e^ | 4.02^***^ [3.77,4.28] | 4.03^***^ [3.77,4.29] | 4.04^***^ [3.78,4.30] | 3.98^***^ [3.60,4.35] | 3.83^***^ [3.54,4.11] | 3.83^***^ [3.54,4.12] | 3.81^***^ [3.52,4.10] | 3.72^***^ [3.30,4.14] |
| Other^e^ | 0.63^***^ [0.48,0.77] | 0.62^***^ [0.48,0.76] | 0.62^***^ [0.48,0.76] | 0.63^***^ [0.43,0.83] | 0.41^***^ [0.29,0.52] | 0.40^***^ [0.29,0.52] | 0.40^***^ [0.29,0.52] | 0.43^***^ [0.27,0.59] |
| Unemployment spending |  | 0.01 [-0.14,0.16] | 0.04 [-0.11,0.19] | 0.01 [-0.15,0.18] |  | -0.07 [-0.23,0.09] | -0.06 [-0.23,0.10] | -0.09 [-0.27,0.09] |
| GDP |  | -0.01^***^ [-0.01,-0.00] | -0.01^***^ [-0.01,-0.00] | -0.01^***^ [-0.01,-0.00] |  | -0.01^***^ [-0.01,-0.00] | -0.01^***^ [-0.01,-0.00] | -0.01^***^ [-0.01,-0.00] |
| Unemployed^e^##unemployment spending |  |  | -0.13 [-0.32,0.05] | 0.10 [-0.21,0.41] |  |  | 0.04 [-0.16,0.24] | 0.01 [-0.32,0.33] |
| Permanently sick/disabled^e^##unemployment spending |  |  | -0.18 [-0.41,0.06] | -0.15 [-0.54,0.25] |  |  | 0.15 [-0.11,0.41] | 0.10 [-0.32,0.51] |
| Other^e^##unemployment spending |  |  | -0.12 [-0.25,0.01] | -0.16 [-0.34,0.01] |  |  | -0.04 [-0.15,0.07] | -0.10 [-0.25,0.05] |
| 2012 survey year^d^##Unemployed^e^ |  |  |  | -0.23 [-0.58,0.11] |  |  |  | -0.21 [-0.60,0.18] |
| 2012 survey year^d^##Permanently sick/disabled^e^ |  |  |  | 0.12 [-0.38,0.63] |  |  |  | 0.16 [-0.41,0.72] |
| 2012 survey year^d^##Other^e^ |  |  |  | 0.00 [-0.26,0.26] |  |  |  | -0.04 [-0.26,0.17] |
| 2012 survey year^d^##Unemployment spending |  |  |  | 0.06 [-0.04,0.17] |  |  |  | 0.04 [-0.08,0.17] |
| 2012 survey year^d^##Unemployed^e^##unemployment spending |  |  |  | -0.39^*^ [-0.78,-0.00] |  |  |  | 0.03 [-0.38,0.44] |
| 2012 survey year^d^##Permanently sick/disabled^e^##unemployment spending |  |  |  | -0.07 [-0.56,0.43] |  |  |  | 0.08 [-0.45,0.61] |
| 2012 survey year^d^##Other^e^##unemployment spending |  |  |  | 0.10 [-0.16,0.35] |  |  |  | 0.11 [-0.10,0.33] |
| Intercept | 4.45^***^ [4.04,4.86] | 6.68^***^ [5.83,7.52] | 6.67^***^ [5.83,7.51] | 6.63^***^ [5.78,7.48] | 4.79^***^ [4.38,5.21] | 6.58^***^ [5.75,7.42] | 6.57^***^ [5.74,7.41] | 6.47^***^ [5.62,7.32] |
| Variance (country) | 0.74 [0.38,1.44] | 0.28^***^ [0.14,0.56] | 0.28^***^ [0.14,0.56] | 0.28^***^ [0.14,0.56] | 0.75 [0.39,1.46] | 0.26^***^ [0.12,0.54] | 0.26^***^ [0.12,0.54] | 0.27^***^ [0.13,0.57] |
| Variance (individual) | 11.96^***^ [11.74,12.18] | 11.95^***^ [11.74,12.17] | 11.95^***^ [11.73,12.17] | 11.95^***^ [11.73,12.17] | 15.08^***^ [14.82,15.34] | 15.08^***^ [14.82,15.35] | 15.08^***^ [14.82,15.35] | 15.08^***^ [14.82,15.34] |
| *N* | 22991 | 22991 | 22991 | 22991 | 25406 | 25406 | 25406 | 25406 |

^a^ Compared to born in country; ^b^ Compared to married/cohabiting with children; ^c^ Compared to high education; ^d^ Compared to 2006 survey wave; ^e^ Compared to employed; ^*^ *p* < 0.05, ^**^ *p* < 0.01, ^***^ *p* < 0.001; ## interaction

Table S5: Linear multilevel models predicting depressive symptoms (CES-D 8) according to education level and ALMP spending among men and women from 18 European countries

|  | **Men** | | | | **Women** | | | |
| --- | --- | --- | --- | --- | --- | --- | --- | --- |
|  | **Model 1** | **Model 2** | **Model 3** | **Model 4** | **Model 1** | **Model 2** | **Model 3** | **Model 4** |
|  | **b**  **[95% CI]** | **b**  **[95% CI]** | **b**  **[95% CI]** | **b**  **[95% CI]** | **b**  **[95% CI]** | **b**  **[95% CI]** | **b**  **[95% CI]** | **b**  **[95% CI]** |
| Age (centered) | 0.01^***^ [0.00,0.01] | 0.01^***^ [0.00,0.01] | 0.01^***^ [0.00,0.01] | 0.01^***^ [0.00,0.01] | 0.02^***^ [0.01,0.02] | 0.02^***^ [0.01,0.02] | 0.02^***^ [0.01,0.02] | 0.02^***^ [0.01,0.02] |
| Age squared | -0.06^***^ [-0.09,-0.02] | -0.06^***^ [-0.09,-0.02] | -0.05^***^ [-0.09,-0.02] | -0.06^***^ [-0.09,-0.02] | -0.04^*^ [-0.07,-0.00] | -0.04^*^ [-0.07,-0.00] | -0.04^*^ [-0.07,-0.00] | -0.04^*^ [-0.07,-0.00] |
| Not born in country^a^ | 0.82^***^ [0.66,0.98] | 0.82^***^ [0.66,0.98] | 0.83^***^ [0.67,0.99] | 0.83^***^ [0.66,0.99] | 0.82^***^ [0.65,0.98] | 0.82^***^ [0.65,0.98] | 0.82^***^ [0.66,0.99] | 0.82^***^ [0.65,0.98] |
| 2012 Survey year^b^ | -0.22^***^ [-0.32,-0.13] | -0.13^*^ [-0.24,-0.03] | -0.14^**^ [-0.25,-0.04] | -0.18 [-0.36,0.01] | -0.29^***^ [-0.39,-0.19] | -0.20^***^ [-0.31,-0.08] | -0.21^***^ [-0.32,-0.10] | -0.19^*^ [-0.37,-0.01] |
| Low education^c^ | 1.22^***^ [1.08,1.36] | 1.21^***^ [1.07,1.35] | 1.26^***^ [1.12,1.40] | 1.13^***^ [0.94,1.33] | 1.78^***^ [1.64,1.92] | 1.78^***^ [1.64,1.92] | 1.79^***^ [1.65,1.93] | 1.83^***^ [1.64,2.03] |
| Medium education^c^ | 0.43^***^ [0.32,0.54] | 0.41^***^ [0.30,0.52] | 0.46^***^ [0.34,0.57] | 0.48^***^ [0.31,0.64] | 0.75^***^ [0.63,0.87] | 0.74^***^ [0.62,0.86] | 0.76^***^ [0.65,0.88] | 0.77^***^ [0.60,0.94] |
| ALMP spending |  | -0.13 [-0.35,0.09] | 0.04 [-0.18,0.27] | 0.09 [-0.17,0.35] |  | 0.05 [-0.17,0.27] | 0.24^*^ [0.01,0.47] | 0.20 [-0.06,0.46] |
| GDP |  | -0.01^***^ [-0.01,-0.00] | -0.01^***^ [-0.01,-0.00] | -0.01^***^ [-0.01,-0.00] |  | -0.01^***^ [-0.01,-0.00] | -0.01^***^ [-0.01,-0.00] | -0.01^***^ [-0.01,-0.00] |
| Low education^c^##ALMP spending |  |  | -0.41^***^ [-0.55,-0.27] | -0.50^***^ [-0.71,-0.30] |  |  | -0.50^***^ [-0.64,-0.36] | -0.70^***^ [-0.90,-0.50] |
| Medium education^c^##ALMP spending |  |  | -0.20^***^ [-0.31,-0.09] | -0.27^***^ [-0.43,-0.11] |  |  | -0.23^***^ [-0.34,-0.11] | -0.27^**^ [-0.43,-0.10] |
| 2012 Survey year^b^##Low education^c^ |  |  |  | 0.26 [-0.01,0.53] |  |  |  | -0.04 [-0.31,0.23] |
| 2012 Survey year^b^##Medium education^c^ |  |  |  | -0.03 [-0.25,0.19] |  |  |  | 0.00 [-0.23,0.23] |
| 2012 Survey year^b^##ALMP spending |  |  |  | -0.07 [-0.24,0.10] |  |  |  | -0.01 [-0.18,0.16] |
| 2012 Survey Year^b^##Low education^c^##ALMP spending |  |  |  | 0.19 [-0.07,0.46] |  |  |  | 0.38^**^ [0.11,0.66] |
| 2012 Survey Year^b^##Medium education^c^##ALMP spending |  |  |  | 0.13 [-0.08,0.35] |  |  |  | 0.07 [-0.15,0.30] |
| Intercept | 4.80^***^ [4.36,5.23] | 6.92^***^ [5.81,8.03] | 6.83^***^ [5.71,7.95] | 6.77^***^ [5.61,7.93] | 5.16^***^ [4.74,5.59] | 7.36^***^ [6.30,8.42] | 7.26^***^ [6.20,8.33] | 7.11^***^ [6.02,8.19] |
| Variance (country) | 0.83 [0.43,1.60] | 0.35^**^ [0.18,0.70] | 0.36^**^ [0.18,0.71] | 0.37^**^ [0.19,0.72] | 0.78 [0.40,1.51] | 0.28^***^ [0.14,0.57] | 0.29^***^ [0.14,0.58] | 0.28^***^ [0.14,0.57] |
| Variance (individual) | 13.07^***^ [12.84,13.31] | 13.06^***^ [12.83,13.30] | 13.04^***^ [12.81,13.28] | 13.04^***^ [12.80,13.28] | 15.95^***^ [15.67,16.23] | 15.95^***^ [15.67,16.23] | 15.92^***^ [15.64,16.19] | 15.91^***^ [15.63,16.19] |
| *N* | 22991 | 22991 | 22991 | 22991 | 25406 | 25406 | 25406 | 25406 |

^a^ Compared to born in country; ^b^ Compared to 2006 survey; ^c^ Compared to high education; ^*^ *p* < 0.05, ^**^ *p* < 0.01, ^***^ *p* < 0.001; ## interaction

Table S6: Linear multilevel models predicting depressive symptoms (CES-D 8) according to education level and ALMP spending among men and women from 18 European countries, controlling for employment status

|  | **Men** | **Women** |
| --- | --- | --- |
|  | **b**  **[95% CI]** | **b**  **[95% CI]** |
| Age (centered) | 0.00 [-0.00,0.01] | 0.02^***^ [0.01,0.02] |
| Age squared | -0.14^***^ [-0.17,-0.10] | -0.06^**^ [-0.09,-0.02] |
| Not born in country^a^ | 0.72^***^ [0.57,0.88] | 0.77^***^ [0.61,0.93] |
| 2012 Survey year^b^ | -0.23^***^ [-0.33,-0.13] | -0.27^***^ [-0.38,-0.16] |
| Low education^c^ | 0.79^***^ [0.65,0.93] | 1.45^***^ [1.31,1.59] |
| Medium education^c^ | 0.30^***^ [0.19,0.41] | 0.64^***^ [0.52,0.76] |
| ALMP spending | 0.13 [-0.08,0.35] | 0.24^*^ [0.02,0.47] |
| Low education^c^##ALMP spending | -0.35^***^ [-0.48,-0.21] | -0.53^***^ [-0.67,-0.39] |
| Medium education^c^##ALMP spending | -0.17^**^ [-0.27,-0.06] | -0.23^***^ [-0.35,-0.12] |
| Unemployed^d^ | 1.94^***^ [1.77,2.11] | 1.46^***^ [1.27,1.65] |
| Permanently sick/disabled^d^ | 4.38^***^ [4.12,4.64] | 4.01^***^ [3.72,4.30] |
| Other^d^ | 0.76^***^ [0.61,0.90] | 0.32^***^ [0.20,0.43] |
| GDP | -0.01^***^ [-0.01,-0.00] | -0.01^***^ [-0.01,-0.00] |
| Intercept | 6.85^***^ [5.80,7.91] | 7.06^***^ [6.01,8.11] |
| Variance (country) | 0.31^***^ [0.16,0.61] | 0.28^***^ [0.13,0.57] |
| Variance (individual) | 12.25^***^ [12.03,12.48] | 15.37^***^ [15.10,15.64] |
| N | 22991 | 25406 |

^a^ Compared to born in country; ^b^ Compared to 2006 survey wave ^c^ Compared to high education;; ^d^ Compared to employed; ^*^ *p* < 0.05, ^**^ *p* < 0.01, ^***^ *p* < 0.001; ## interaction

Table S7: Linear multilevel models predicting depressive symptoms (CES-D 8) according to family status and family spending among men and women from 18 European countries

|  | **Men** | | | | **Women** | | | |
| --- | --- | --- | --- | --- | --- | --- | --- | --- |
|  | **Model 1** | **Model 2** | **Model 3** | **Model 4** | **Model 1** | **Model 2** | **Model 3** | **Model 4** |
|  | **b**  **[95% CI]** | **b**  **[95% CI]** | **b**  **[95% CI]** | **b**  **[95% CI]** | **b**  **[95% CI]** | **b**  **[95% CI]** | **b**  **[95% CI]** | **b**  **[95% CI]** |
| Age (centered) | 0.03^***^ [0.02,0.03] | 0.03^***^ [0.02,0.03] | 0.03^***^ [0.02,0.03] | 0.03^***^ [0.02,0.03] | 0.02^***^ [0.02,0.03] | 0.02^***^ [0.02,0.03] | 0.02^***^ [0.02,0.03] | 0.02^***^ [0.02,0.03] |
| Age squared | -0.18^***^ [-0.21,-0.14] | -0.18^***^ [-0.21,-0.14] | -0.18^***^ [-0.21,-0.14] | -0.18^***^ [-0.21,-0.14] | -0.12^***^ [-0.16,-0.08] | -0.12^***^ [-0.16,-0.08] | -0.12^***^ [-0.16,-0.08] | -0.12^***^ [-0.16,-0.08] |
| Not born in country^a^ | 0.92^***^ [0.76,1.08] | 0.92^***^ [0.76,1.08] | 0.91^***^ [0.75,1.07] | 0.92^***^ [0.76,1.08] | 0.82^***^ [0.66,0.99] | 0.82^***^ [0.66,0.99] | 0.82^***^ [0.65,0.98] | 0.82^***^ [0.65,0.98] |
| Low education^b^ | 1.12^***^ [0.98,1.26] | 1.12^***^ [0.98,1.25] | 1.11^***^ [0.98,1.25] | 1.11^***^ [0.98,1.25] | 1.77^***^ [1.63,1.91] | 1.77^***^ [1.63,1.91] | 1.77^***^ [1.63,1.91] | 1.77^***^ [1.63,1.91] |
| Medium education^b^ | 0.38^***^ [0.27,0.49] | 0.36^***^ [0.25,0.47] | 0.36^***^ [0.25,0.47] | 0.36^***^ [0.25,0.47] | 0.73^***^ [0.61,0.85] | 0.72^***^ [0.61,0.84] | 0.72^***^ [0.61,0.84] | 0.72^***^ [0.61,0.84] |
| 2012 Survey year^c^ | -0.26^***^ [-0.35,-0.17] | -0.17^**^ [-0.28,-0.06] | -0.17^**^ [-0.28,-0.05] | -0.15 [-0.31,0.01] | -0.32^***^ [-0.42,-0.23] | -0.21^***^ [-0.33,-0.09] | -0.21^***^ [-0.33,-0.10] | -0.14 [-0.31,0.03] |
| Married/cohabiting without children^d^ | 0.22^***^ [0.09,0.34] | 0.22^***^ [0.09,0.34] | 0.23^***^ [0.11,0.36] | 0.15 [-0.02,0.32] | -0.00 [-0.14,0.13] | 0.00 [-0.13,0.14] | 0.01 [-0.13,0.14] | 0.04 [-0.15,0.22] |
| Single with children^d^ | 1.91^***^ [1.59,2.24] | 1.91^***^ [1.58,2.23] | 2.02^***^ [1.69,2.36] | 2.00^***^ [1.53,2.48] | 1.58^***^ [1.41,1.75] | 1.58^***^ [1.41,1.75] | 1.58^***^ [1.41,1.75] | 1.75^***^ [1.50,2.00] |
| Single without children^d^ | 1.66^***^ [1.54,1.78] | 1.66^***^ [1.54,1.78] | 1.66^***^ [1.54,1.78] | 1.82^***^ [1.65,1.99] | 1.21^***^ [1.07,1.35] | 1.21^***^ [1.07,1.35] | 1.22^***^ [1.08,1.36] | 1.27^***^ [1.07,1.47] |
| Family spending |  | 0.06 [-0.18,0.31] | 0.06 [-0.19,0.31] | 0.18 [-0.15,0.50] |  | -0.12 [-0.36,0.11] | -0.07 [-0.32,0.17] | -0.19 [-0.51,0.14] |
| GDP |  | -0.01^***^ [-0.01,-0.01] | -0.01^***^ [-0.01,-0.01] | -0.01^***^ [-0.01,-0.01] |  | -0.01^***^ [-0.01,-0.00] | -0.01^***^ [-0.01,-0.00] | -0.01^***^ [-0.01,-0.00] |
| Married/cohabiting without children^d d^##Family spending |  |  | -0.11 [-0.23,0.01] | -0.11 [-0.29,0.07] |  |  | -0.15^*^ [-0.27,-0.02] | -0.18 [-0.37,0.02] |
| Single with children^d^ ##Family spending |  |  | -0.48^**^ [-0.80,-0.16] | -0.63^*^ [-1.14,-0.12] |  |  | -0.28^***^ [-0.44,-0.11] | -0.31^*^ [-0.57,-0.04] |
| Single without children^d^ ##Family spending |  |  | 0.11 [-0.00,0.22] | 0.27^**^ [0.10,0.44] |  |  | 0.09 [-0.03,0.22] | 0.23^*^ [0.03,0.44] |
| 2012 Survey year^c^## Married/cohabiting without children ^d^ |  |  |  | 0.17 [-0.07,0.40] |  |  |  | -0.06 [-0.30,0.19] |
| 2012 Survey year^c^## Single with children^d^ |  |  |  | 0.01 [-0.66,0.68] |  |  |  | -0.33 [-0.67,0.01] |
| 2012 Survey year^c^## Single without children^d^ |  |  |  | -0.27^*^ [-0.49,-0.05] |  |  |  | -0.06 [-0.32,0.20] |
| 2012 Survey year^c^##Family spending |  |  |  | -0.01 [-0.17,0.14] |  |  |  | 0.09 [-0.08,0.25] |
| 2012 Survey year^c^## Married/cohabiting without children^d d^##Family spending |  |  |  | -0.02 [-0.26,0.22] |  |  |  | 0.05 [-0.20,0.31] |
| 2012 Survey year^c^## Single with children^d^ ##Family spending |  |  |  | 0.23 [-0.43,0.89] |  |  |  | 0.09 [-0.25,0.43] |
| 2012 Survey year^c^## Single without children^d^ ##Family spending |  |  |  | -0.23^*^ [-0.46,-0.01] |  |  |  | -0.22 [-0.49,0.04] |
| Intercept | 4.43^***^ [4.01,4.86] | 6.98^***^ [6.08,7.87] | 6.97^***^ [6.08,7.86] | 7.32^***^ [6.31,8.33] | 4.88^***^ [4.47,5.30] | 6.66^***^ [5.78,7.53] | 6.68^***^ [5.80,7.55] | 6.41^***^ [5.41,7.42] |
| Variance (country) | 0.78 [0.40,1.52] | 0.31^***^ [0.16,0.61] | 0.31^***^ [0.15,0.61] | 0.30^***^ [0.15,0.59] | 0.74 [0.38,1.45] | 0.26^***^ [0.13,0.54] | 0.26^***^ [0.13,0.53] | 0.28^***^ [0.13,0.58] |
| Variance (individual) | 12.59^***^ [12.37,12.83] | 12.59^***^ [12.36,12.82] | 12.57^***^ [12.35,12.81] | 12.56^***^ [12.34,12.80] | 15.57^***^ [15.30,15.84] | 15.57^***^ [15.30,15.84] | 15.56^***^ [15.29,15.83] | 15.55^***^ [15.28,15.82] |
| *N* | 22991 | 22991 | 22991 | 22991 | 25406 | 25406 | 25406 | 25406 |

^a^ Compared to born in country; ^b^ Compared to high education; ^c^ Compared to 2006 survey wave; ^d^ Compared to married/cohabiting with children

^*^ *p* < 0.05, ^**^ *p* < 0.01, ^***^ *p* < 0.001; ; ## interaction
